# Including uncertainty of the expected mortality rates in the prediction of loss in life expectancy

**DOI:** 10.1186/s12874-023-02118-w

**Published:** 2023-12-12

**Authors:** Yuliya Leontyeva, Mats Lambe, Hannah Bower, Paul C. Lambert, Therese M.-L. Andersson

**Affiliations:** 1 Department of Medical Epidemiology and Biostatistics, Karolinska Institutet, Stockholm, Sweden; 2 Clinical Epidemiology Division, Department of Medicine Solna, Karolinska Institutet, Stockholm, Sweden; 3 Department of Population Health Sciences, Biostatistics research group, University of Leicester, Leicester, UK

**Keywords:** Relative survival, Expected mortality rates, Loss in life expectancy, Flexible parametric survival models

## Abstract

**Purpose:**

This study introduces a novel method for estimating the variance of life expectancy since diagnosis (LE_C_) and loss in life expectancy (LLE) for cancer patients within a relative survival framework in situations where life tables based on the entire general population are not accessible. LE_C_ and LLE are useful summary measures of survival in population-based cancer studies, but require information on the mortality in the general population. Our method addresses the challenge of incorporating the uncertainty of expected mortality rates when using a sample from the general population.

**Methods:**

To illustrate the approach, we estimated LE_C_ and LLE for patients diagnosed with colon and breast cancer in Sweden. General population mortality rates were based on a random sample drawn from comparators of a matched cohort. Flexible parametric survival models were used to model the mortality among cancer patients and the mortality in the random sample from the general population. Based on the models, LE_C_ and LLE together with their variances were estimated. The results were compared with those obtained using fixed expected mortality rates.

**Results:**

By accounting for the uncertainty of expected mortality rates, the proposed method ensures more accurate estimates of variances and, therefore, confidence intervals of LE_C_ and LLE for cancer patients. This is particularly valuable for older patients and some cancer types, where underestimation of the variance can be substantial when the entire general population data are not accessible.

**Conclusion:**

The method can be implemented using existing software, making it accessible for use in various cancer studies. The provided example of Stata code further facilitates its adoption.

**Supplementary Information:**

The online version contains supplementary material available at 10.1186/s12874-023-02118-w.

## Introduction

The loss in life expectancy (LLE) for cancer patients, or the number of years lost due to cancer, is a useful complementary measure for summarising cancer prognosis. LLE gives an understanding of the impact of a cancer diagnosis over the whole life span, can be used on both an individual and on a population level and is easy to communicate. LLE is calculated as the difference between the life expectancy of cancer patients (LE_C_) and the life expectancy they would have if they had not been diagnosed with cancer (LE_exp_). The latter, expected life expectancy of a cancer patient (LE_exp_), is approximated by the life expectancy of an individual from the general population of the same age, sex and potentially other factors. LE_C_ is obtained as the area under the observed (all-cause) survival curve for cancer patients and is usually estimated in the relative survival framework [1]. The relative survival framework is often used for estimating cancer patient survival in population-based studies because it does not require information on cause of death. Within the relative survival framework, the all-cause survival for cancer patients is presented as the product of the survival a cancer patient would experience if they did not have cancer, or expected survival, and relative survival (RS). Under certain assumptions, RS is an estimate of net survival, which represents the survival in absence of other causes of death. The expected survival is assumed to be the same as the survival of matched individuals in the general population given a specific covariate pattern and, in practice, usually obtained from general population life tables, and so is LE_exp_ described above. Any uncertainty in the estimates of the expected survival and expected mortality (an analogue to the expected survival on the hazard scale) obtained from general population life tables is assumed to be negligible. Thus, the expected survival, expected mortality and LE_exp_ for cancer patients are treated as known or fixed values and do not contribute to the variability of LE_C_ and LLE.

Recent work has shown that when estimating RS and LLE for colon cancer patients in Sweden using life tables from the entire general population, uncertainty in the expected survival and mortality can often be ignored when calculating SEs [2]. Here, the entire general population refers to all people living in a country or region, i.e. the catchment area for the population-based cancer registry. However, it has also been illustrated that in some situations the uncertainty in the expected survival and mortality should be taken into account, otherwise, SEs for RS and LLE will be too small and confidence intervals too narrow [2]. These situations may include, but not limited to, when estimates of the expected measures are not based on the entire general population but on a sample from the general population.

The need for the sample may arise when certain characteristics, which may affect expected mortality rates, are unavailable at the population level. This situation also becomes necessary in cancer randomised trials, where participants are carefully selected based on specific inclusion criteria. In both scenarios, estimating the expected mortality for cancer patients using general population data might be not appropriate. It has been demonstrated that using mismatched life tables can introduce biases in estimating excess mortality [3]. While this issue, known as non-comparability bias, has received extensive attention in the literature [4–7], there remains a gap in addressing the variability of expected values.

Moreover, the impact of ignoring the uncertainty in the expected survival or the expected mortality has a more substantial effect on the estimates of SE of LLE than SE of RS [2]. This is due to the fact that the expected mortality rates are included in several parts of the estimation of LLE, namely, the estimation of life expectancy in the general population and life expectancy of the cancer patients, which in turn, is estimated using the expected mortality rates and excess mortality rates.

In a previous study [2], the necessity of incorporating uncertainty in the expected measures was evaluated under various scenarios. To conduct this assessment, a parametric bootstrap approach was employed. This involved generating 1000 realisations of general population mortality rates and obtaining 1000 estimates. Such an approach can be computationally intensive and time-consuming, potentially not very practical. The aim of this study is to develop an approach to incorporate the uncertainty of the expected measures in the estimation of LLE when a sample from the general population is used for the estimation of expected measures. The approach is illustrated using data on breast and colon cancer in Sweden. The proposed method has the advantage of using existing Stata software.

The remainder of this paper is laid out as follow. “Background” section describes an existing approach of estimation of LE_C_ and LLE when uncertainty in estimates of the expected measures is ignored. “Estimation of LE_exp_ including uncertainty in the expected survival and mortality of the general population”, “Estimation of LE_C_ including uncertainty in the expected survival and mortality of the general population” and “Estimation of Var(LLE) including uncertainty in the expected survival and mortality of the general population” sections describe how uncertainty of the expected measures can be incorporated in estimation of LE_exp_, LE_C_ and LLE, respectively. In “Data and analysis” section we present the data used in the analysis. “Results” section presents the results and compares estimates obtained when uncertainty in the expected measures is included with the estimates when uncertainty in the expected measures is ignored. Finally, “Discussion” section discusses the proposed method.

## Methods

### Background

Life expectancy is a well-known concept quantifying the average number of years an individual is expected to live. For cancer patients, life expectancy (LE_C_) quantifies the average number of life years remaining at diagnosis, while the loss in life expectancy (LLE) is the average number of life years a cancer patient loses due to cancer. The LLE for cancer patients is estimated as the difference between the life expectancy cancer patients would have if they did not have cancer LE_exp_ and the life expectancy of cancer patients LE_C_:

$$\begin{aligned} LLE (Z) = LE_{\text {exp}}(Z_2) - LE_C(Z), \end{aligned}$$

where $LE_{\text {exp}}(Z_2)$ and $LE_{C}(Z)$ are calculated as the area under the corresponding survival curve, the survival cancer patients would have if they did not have cancer $S^*(t | Z_2)$ (also referred to as expected survival) and the observed (all-cause) survival for cancer patients *S*(*t*|*Z*), respectively:

$$\begin{aligned} LE_{\text {exp}} (Z_2) = \int _0^{t^*} S^*(u | Z_2) du \\ LE_C (Z) = \int _0^{t^*} S(u | Z) du \end{aligned}$$

Using these, the above equation for LLE becomes:

1 $$\begin{aligned} LLE (Z) = \int _0^{t^*} S^*(u | Z_2) du - \int _0^{t^*} S(u | Z) du, \end{aligned}$$

where $t^*$ is the maximum time when both survival curves, the expected survival $S^*(t|Z_2)$ and all-cause survival *S*(*t*|*Z*) are effectively zero. $Z_2$ denotes a set of covariates for the life expectancy of cancer patients if they did not have cancer while *Z* presents the covariates for the life expectancy of cancer patients at cancer diagnosis and includes $Z_2$.

In practice, expected survival $S^*(t|Z_2)$ is assumed to be the same as the survival in the general population, obtained from the general population life tables stratified by some sociodemographic covariates $Z_2$ like age, gender and calendar year. The uncertainty in the estimates of expected measures based on the entire general population, i.e. all people living in a country, region or the catchment area for the population-based cancer registry, is negligible with regards to the uncertainty in a much smaller cancer population and is, therefore, usually ignored [2].

Estimation of $LE_C (Z)$ most often requires extrapolation of *S*(*t*|*Z*) in the cancer cohort beyond the study period since follow-up until the death of all cancer patients, i.e. until the observed survival curve is effectively zero, is not feasible. For most cancer types this extrapolation has been shown to perform well in a relative survival framework [8]. Within the relative survival framework the all-cause mortality rate for cancer patients, *h*(*t*|*Z*), can be partitioned into the mortality rate due to cancer, and the mortality rate due to other causes. The mortality rate due to other causes is assumed to be the same as the mortality rate of an individual in the general population, matched on age, sex, calendar year and possibly other covariates, and referred to as expected mortality, $h^*(t |Z_2)$, and the mortality due to cancer is referred to as excess mortality $\lambda (t | Z_1)$, the mortality rate in excess to the expected mortality. $Z_1$ presents covariates for the cancer-specific death and *Z* is the combination of $Z_1$ and $Z_2$. Very often *Z*, $Z_1$ and $Z_2$ will be the same. The extrapolation of the all-cause mortality is performed separately for the expected and excess mortality rates. On a survival scale, all-cause survival for cancer patients *S*(*t*|*Z*) is the product of expected survival $S^*(t |Z_2)$ and relative survival $R(t | Z_1)$:

2 $$\begin{aligned} S(t | Z) = R(t | Z_1) \cdot S^*(t | Z_2) \end{aligned}$$

The relative survival can be estimated from a flexible parametric relative survival model (FPRM) [9]. The log cumulative excess hazard $\ln {[\Lambda (t|Z_1)]}$ within a FPRM is expressed as:

3 $$\begin{aligned} \ln [\Lambda (t| Z_1)] = s(\ln (t)|\varvec{\gamma _1},\varvec{k_1}) + \varvec{\beta _1 Z_1}, \end{aligned}$$

where *t* represents time since cancer diagnosis, $s(\ln (t)|\varvec{\gamma _1},\varvec{k_1})$ is a restricted cubic spline function of $\ln (t)$ used to estimate the baseline log cumulative excess hazard [10], $\varvec{Z_1}$ represents a vector of covariates for excess mortality. Model (3) is a proportional excess hazards model but time-dependent effects can be incorporated by including interactions between covariates and a spline function of log time [11]. The estimates of parameters ($\widehat{\varvec{\beta _1}}$, $\widehat{\varvec{\gamma _1}}$) from Model (3) are obtained using maximum likelihood, where the contribution of the *i*-th individual to the log-likelihood *l* can be written as:

$$\begin{aligned}{} & {} l_i (\varvec{\beta _1}, \varvec{\gamma _1} | t_i, \varvec{Z_i}) = d_i\ln [h^*(t_i | \varvec{Z_{2_i}} ) + \lambda (t_i | \varvec{Z_{1_i}}, \varvec{\beta _1}, \varvec{\gamma _1})] + \ln [S^*(t_i | \varvec{Z_{2_i}})]\\{} & {} + \ln [R(t_i | \varvec{Z_{1_i}}, \varvec{\beta _1}, \varvec{\gamma _1})], \end{aligned}$$

where $d_i$ is the death indicator.

We assume that $h^*(t | Z_2)$ and $S^*(t | Z_2)$ are known, i.e. measured without uncertainty. As they do not depend on the model parameters, $S^*(t | Z_2)$ can be dropped from the log-likelihood and *l* can be rewritten as:

4 $$\begin{aligned} l_i (\varvec{\beta _1}, \varvec{\gamma _1} | t_i, \varvec{Z_i}) = d_i\ln [h^*(t_i | \varvec{Z_{2_i}}) + \lambda (t_i | \varvec{Z_{1_i}}, \varvec{\beta _1}, \varvec{ \gamma _1})] + \ln [R(t_i | \varvec{Z_{1_i}}, \varvec{\beta _1}, \varvec{ \gamma _1})] \end{aligned}$$

Here, for each cancer patient, *i*, their expected mortality rate, $h^*(t_i | Z_2)$, given covariates $Z_2$ at the time of death due to any cause, $t_i$, is assumed to be known, and most often obtained from life tables based on the entire general population. We denote a variance-covariance matrix of $\widehat{\varvec{\beta _1}}$ and $\widehat{\varvec{\gamma _1}}$ as $V_1$.

Using estimates from Model (3) and the relationship between the cumulative hazard function and survival function, $\widehat{R}(t | Z_1)$ can be obtained by

5 $$\begin{aligned} \widehat{R}(t|Z_1)=\exp \left( -\exp (\ln [\widehat{\Lambda }(t|Z_1)])\right) \end{aligned}$$

LLE can be estimated in the relative survival setting as

6 $$\begin{aligned} \widehat{LLE}(Z) = \int _0^{t^*} S^*(u|Z_2) du - \int _0^{t^*} \widehat{R}(u|Z_1) \cdot S^*(u|Z_2) du, \end{aligned}$$

Since $S^*(t|Z_2)$ is treated as fixed, it does not contribute to the variance of LLE, i.e.:

7 $$\begin{aligned}{} & {} Var(\widehat{LLE}) = Var(\widehat{LE_{\text {exp}}} - \widehat{LE_C}) = Var(\widehat{LE_{\text {exp}}}) + Var(\widehat{LE_C}) -2\cdot COV(\widehat{LE_{\text {exp}}},\widehat{LE_C}) \nonumber \\{} & {} = Var(\widehat{LE_C}) = Var \left[ \int _0^{t^*} \widehat{R}(u|Z_1) \cdot S^*(u|Z_2) du \right] \nonumber \\{} & {} = Var\left[ \int _0^{t^*} \exp (-\exp (\ln [\widehat{\Lambda }(u|Z_1)]))\cdot S^*(u|Z_2) du \right] , \end{aligned}$$

which can be obtained using the delta method [12]. In this case, the uncertainty of the LLE solely comes from the uncertainty in excess mortality.

In situations, where there may be concerns about the extrapolation of survival curves, for example, for young cancer patients, or for long follow-up times, restricted mean survival times (RMST) can be obtained [13]. Expected restricted mean survival time (RMST_exp_), observed restricted mean survival time for cancer patients (RMST_C_) and the difference (loss) between restricted mean survival times (LRMST) for cancer patients are estimated within a predefined time window.

### Estimation of LE_exp_ including uncertainty in the expected survival and mortality of the general population

It has been shown that the uncertainty in the expected survival should be taken into account when the estimates are based on a sample from the general population [2]. An example of such a sample can be comparators from a matched cohort study, where cancer patients are matched on age to comparators from the general population. By fitting a survival model to estimate mortality for the comparators, the predicted rates can be used as an alternative for $h^*(t | Z_2)$, and the uncertainty of the estimates can be obtained.

We suggest using a flexible parametric survival model (FPM) [14] with attained age as a time-scale to estimate the mortality rate for the comparators:

8 $$\begin{aligned} \ln [H(a|Z_2)] = s(\ln (a)|\varvec{\gamma _2},\varvec{k_2}) + \varvec{\beta _2 Z_2}, \end{aligned}$$

where *a* is the attained age, $\varvec{Z_2}$ is a vector of covariates for the expected survival, $H(a|Z_2)$ is the cumulative expected hazard, $s(\ln (a)|\varvec{\gamma _2},\varvec{k_2})$ is a restricted cubic spline function of $\ln (a)$, used to estimate the baseline log cumulative hazard. Model (8) is a proportional hazards model but can easily be extended to non-proportional hazards by incorporating interactions between covariates and spline terms for $\ln (a)$.

Parameter estimates $\widehat{\varvec{\beta _2}}$ and $\widehat{\varvec{\gamma _2}}$ from Model (8) are obtained by maximum likelihood that incorporates the potential delayed entry (left-truncation) and can be written as follows:

$$\begin{aligned}{} & {} l_i (\varvec{\beta _2}, \varvec{\gamma _2} | a_{0_i}, a_i, \varvec{Z_{2_i}}) = d_i\ln [h^*(a_i | \varvec{Z_{2_i}}, \varvec{\beta _2}, \varvec{\gamma _2})] + \ln [S^*(a_i | \varvec{Z_{2_i}}, \varvec{\beta _2}, \varvec{\gamma _2})] \\{} & {} - \ln [S^*(a_{0_i} | \varvec{Z_{2_i}}, \varvec{\beta _2}, \varvec{\gamma _2})] , \end{aligned}$$

where $a_{0_i}$ is the age at the beginning of the follow-up period for *i*-th individual.

Using the general relationship between cumulative hazard, hazard and survival, $\widehat{S^*}(a)$ can be obtained by:

9 $$\begin{aligned} \widehat{S^*}(a|Z_2) = \exp \left( -\exp (\ln [\widehat{H}(a|Z_2)])\right) \end{aligned}$$

Then $\widehat{LE_{\text {exp}}}(Z_2)$ with attained age as time scale is estimated as:

10 $$\begin{aligned} \widehat{LE_{\text {exp}}}(Z_2) = \int _{a_0}^{a_0 + t^*} \frac{\widehat{S^*}(u' | Z_2) }{\widehat{S^*}(a_0 | Z_2)} du', \end{aligned}$$

where $t^*$ is the maximum of follow-up time when everyone is expected to have died and $a_0$ is the age at matching (age at diagnosis for the corresponding matched cancer patient). We can rewrite Eq. (10) with time since diagnosis as time scale by taking into account that attained age is a function of time, i.e.: $a = a_0 + t$. Then by putting $u = u' - a_0$, we rewrite:

11 $$\begin{aligned} \widehat{LE_{\text {exp}}}(Z_2) = \int _{a_0}^{a_0 + t^*} \frac{\widehat{S^*}(u' | Z_2) }{\widehat{S^*}(a_0 | Z_2)} du' = \int _0^{t^*} \widehat{S^*}(u + a_0 | Z_2, a_0) du. \end{aligned}$$

The variance of $\widehat{LE_{\text {exp}}}$ can be obtained using the delta method:

12 $$\begin{aligned} Var(\widehat{LE_{\text {exp}}})=G_E^T \cdot V_2 \cdot G_E \end{aligned}$$

where $V_2$ is the variance-covariance matrix for $\widehat{\varvec{\beta _2}}$ and $\widehat{\varvec{\gamma _2}}$ from Model (8) and $\varvec{G_E}$ is a vector of the first derivatives of function LE_exp_ (Eq. (11)) with respect to each of the parameters $\varvec{\beta _2}$ and $\varvec{\gamma _2}$.

### Estimation of LE_C_ including uncertainty in the expected survival and mortality of the general population

Recall, that $LE_C(Z) = \int _0^{t^*} R(u | Z_1) \cdot S^*(u | Z_2) du$ (Eq. (6)). By using the estimates of $\widehat{R}(t|Z_1)$ from Model (3) and the estimates of $\widehat{S^*}(t|Z_2)$ from Model (8), $LE_C(Z)$ can be written:

13 $$\begin{aligned}{} & {} \widehat{LE_C}(Z) = \int _0^{t^*} \widehat{R}(u | Z_1) \cdot \widehat{S^*}(u + a_0 | Z_2) du \nonumber \\{} & {} = \int _0^{t^*} \exp \left( -\exp (\ln [\widehat{\Lambda }(u|Z_1)])\right) \cdot \exp \left( -\exp (\ln [\widehat{H}(u + a_0 | Z_2])\right) du, \end{aligned}$$

where $\Lambda (t | Z_1)$ is the cumulative excess mortality, while $H(t + a_0 | Z_2)$ is the cumulative expected mortality.

The relative survival *R*(*t*) is interpreted as net survival, i.e. survival from specific cancer in a hypothetical world where a cancer patient can die only from the cancer of interest if conditional independence assumption holds. In other words, conditional on covariates cancer-specific mortality and mortality due to other causes, are independent [15]. They are competing but mutually exclusive events. Therefore, for implementation purposes to use existing Stata software, Model (13) can be specified in terms of a competing risks approach [16], where all-cause survival *S*(*t*) can be presented as:

$$\begin{aligned} S(t) = 1 - \left( Cr_{cancer}(t) + Cr_{other}(t)\right) \end{aligned}$$

Here, $Cr_{cancer}(t)$ is the crude probability of death due to cancer, interpreted as the probability of dying from cancer by time *t*, while also being at risk of dying from other causes and $Cr_{other}(t)$ is the crude probability of death due to other causes interpreted as the probability of dying due to other than the cancer of interest causes by time *t*, while at risk of the cancer death [17]. It should be noted that the notation crude probability of death is used in the relative survival framework, while it is also known as cumulative incidence function in competing risk terminology [18]. Crude probability of death due to cancer and crude probability of death due to other causes can be estimated as:

$$\begin{aligned}{} & {} \widehat{Cr}_{cancer} (t|Z) = \int _0^{t^*} \widehat{S^*}(u + a_0|Z_2) \cdot \widehat{R}(u|Z_1) \cdot \widehat{\lambda }(u|Z_1) du \nonumber \\{} & {} \widehat{Cr}_{other} (t|Z) = \int _0^{t^*} \widehat{S^*}(u + a_0|Z_2) \cdot \widehat{R}(u|Z_1) \cdot \widehat{h^*}(u|Z_2) du \end{aligned}$$

The life expectancy for cancer patients is then estimated as:

$$\begin{aligned} \widehat{LE_C}(Z) = \int _0^{t^*} S(u | Z) du = t^* - \int _0^{t^*} (\widehat{Cr}_{cancer}(u |Z) + \widehat{Cr}_{other}(u|Z)) du, \end{aligned}$$

where $t^*$ is a pre-defined time point after cancer diagnosis when we expect all individuals to have died. This use of the competing risk approach (i.e. by re-writing LE_C_ with respect to $Cr_s$) allows us to use the Stata command standsurv [19] to obtain $\widehat{LE_C}$, its SE and a vector of the first partial derivatives for the function $\widehat{LE_C}$ with respect to each parameter from both models (3) and (8), i.e. with respect to vector $(\varvec{\beta _1, \gamma _1, \beta _2, \gamma _2})^T$. We denote this vector of the first partial derivatives $\varvec{G_C}$.

### Estimation of Var(LLE) including uncertainty in the expected survival and mortality of the general population

Recall, that loss in life expectancy is obtained as the difference between life expectancy for cancer patients and their life expectancy if they did not have cancer. Therefore, to get the variance of LLE, we need to know the variance of LE_exp_, the variance of LE_C_ and their covariance (Eq. 7). $Var(\widehat{LE_{\text {exp}}})$ is obtained as shown in Eq. (12). $Var(\widehat{LE_C})$ is obtained as described above.

To obtain $Cov(\widehat{LE_{\text {exp}}}, \widehat{LE_C})$, let $\varvec{G}$ denote a matrix of observation-specific first derivatives for $\widehat{LE_{\text {exp}}}$ and $\widehat{LE_C}$ with respect to each of parameters from both model (8) and model (3), i.e. with respect to $(\varvec{\beta _1, \gamma _1, \beta _2, \gamma _2})^T$:

$$\begin{aligned} \varvec{G} = \left( \begin{array}{c} \varvec{G_E^*}\\ \varvec{G_C} \end{array}\right) \end{aligned}$$

Note that $\varvec{G_E^*}$ is a vector of observation-specific first derivatives for $\widehat{LE_{\text {exp}}}$ with respect to $(\beta _1, \gamma _1, \beta _2, \gamma _2)^T$, i.e. $\varvec{G_E^*}$ includes $\varvec{G_E}$, a vector of the first derivatives for $\widehat{LE_{\text {exp}}}$ with respect to parameters ($\varvec{\beta _2}, \varvec{\gamma _2}$) and a vector of $\varvec{0_s}$, a vector of the first derivatives for $\widehat{LE_{\text {exp}}}$ with respect to parameters ($\varvec{\beta _1}, \varvec{\gamma _1}$) because models (8) and (3) do not have shared parameters.

Let $\varvec{V}$ denote a combination of two variance-covariance matrices $\varvec{V_1}$ and $\varvec{V_2}$ from models (3) and (8), respectively:

$$\begin{aligned} \varvec{V} = \left( \begin{array}{cc} \varvec{V_2} &{} \varvec{0} \\ \varvec{0} &{} \varvec{V_1} \end{array}\right) \end{aligned}$$

Note that $\varvec{0}$s in $\varvec{V}$ convey that models (3) and (8) do not have shared parameters.

And let $\varvec{\Sigma }$ be a result of matrix multiplication:

14 $$\begin{aligned} \varvec{\Sigma } = \varvec{G^T} \cdot \varvec{V} \cdot \varvec{G}, \end{aligned}$$

where $\varvec{\Sigma }$ can be rewritten:

15 $$\begin{aligned} \varvec{\Sigma } = \left( \begin{array}{cc} \sigma ^2_{\widehat{LE_{\text {exp}}}} &{} \sigma _{\widehat{LE_{\text {exp}}},\widehat{LE_C}} \\ &{}\\ \sigma _{\widehat{LE_C},\widehat{LE_{\text {exp}}}} &{} \sigma ^2_{\widehat{LE_C}} \end{array}\right) . \end{aligned}$$

The estimates from Matrix (15) are used to calculate $Var(\widehat{LLE})$.

### Marginal estimates including uncertainty in the expected survival and mortality of the general population

For population-based cancer studies, it is common to estimate marginal measures to summarise survival in the cancer population. The appealing feature of the marginal estimates is that they have a simple interpretation even though the underlying models are complex, and provide estimates on the population level [20]. To obtain marginal estimates of LE_exp_, LE_C_ and LLE we use regression standardisation. For all individuals in the cancer population, we predict LE_exp_, LE_C_ and LLE and average them by taking the mean for the *N* individuals in the cancer cohort [21]:

$$\begin{aligned}{} & {} \widehat{LE}_{\textrm{exp}_{\textrm{m}}} = \frac{1}{N} \sum \limits _{i=1}^{N} \widehat{LE}_{\textrm{exp}_{\textrm{i}}}(Z_{2_i}) \\{} & {} \widehat{LE}_{C_m} = \frac{1}{N} \sum \limits _{i=1}^N \widehat{LE}_{C_i}(Z_i) \\{} & {} \widehat{LLE}_m = \frac{1}{N} \sum \limits _{i=1}^N \widehat{LLE}_i(Z_i), \end{aligned}$$

where $\widehat{LE}_{\textrm{exp}_{\textrm{i}}}(Z_{2_i})$, $\widehat{LLE}_i(Z_i)$ and $\widehat{LE}_{C_i}(Z_i)$ are the predicted estimates for individual *i* from the cancer cohort. The variance of marginal LLE is obtained in the same way as described above.

A more detailed description of the calculation of the variance of LLE can be found in Supplementary file 7 and an example Stata code is provided in Supplementary files 5 and 6.

## Data and analysis

### Data

In this study, we used Breast Cancer Data Base Sweden (BcBase2), a Swedish Quality breast cancer database, which includes information on women diagnosed with breast cancer in the health care regions of Central Sweden (Uppsala-Örebro), Stockholm-Gotland and Northern Sweden. The data set also includes age-matched controls without breast cancer at matching. To investigate whether including uncertainty in the prediction of LLE would differ for different cancer types, we also used data from the Swedish Cancer Registry to identify women diagnosed with colon cancer in the Central, Stockholm-Gotland and Northern regions of Sweden.

Only women diagnosed with invasive breast cancer were included in the breast cancer cohort. In both cohorts we identified women diagnosed at age 50 or older in the years 1992 to 2003 in the same regions. The breast cancer patients were followed from the date of diagnosis until death, the date of censoring due to first emigration or the end of follow-up (December 31st 2014); whichever occurred first but for a maximum of 15 years. In total, 25,927 breast cancer patients were included in this study. The colon cancer patients were followed from the date of diagnosis until death or the end of follow-up (December 31st 2017); whichever occurred first but for a maximum of 15 years. In total, 9,114 colon cancer patients were included.

This study was approved by the Swedish Ethical Review Authority. Informed consent from study subjects was not required for the current study. This study was carried out in accordance with the Declaration of Helsinki, and all methods were carried out in accordance with relevant guidelines and regulations in Sweden.

### Analysis

To imitate the situation, when life tables based on the entire general population are unavailable, and only a small sample is at hand, a random sample of 5,000 individuals was drawn from the matched comparators included in BcBase2. The choice of this sample size was justified by findings from a previous study [2]. There it was shown that when using the entire general population (i.e., the catchment area for the population-based cancer registry) or a sufficiently large part of the general population to estimate expected mortality and expected survival, uncertainty in estimates of the expected values was fairly negligible. However, when estimating expected values based on the general population reduced to 0.5% of its original size, which is approximately 5,000 individuals for the breast cancer cohort, accounting for uncertainty in these estimates became necessary. While the sample was drawn from matched comparators for the breast cancer patients, it can also be employed to estimate age- and calendar year-specific expected mortality rates for colon cancer patients, assuming no other influential factors on expected mortality rates for the cancer patients.

A FPM as described in Eq. (8) was fitted, where the baseline log cumulative hazard was modelled smoothly using restricted cubic splines with 5 degrees of freedom (df). The calendar year of matching (i.e. the year of diagnosis for the breast cancer patient) was included in the model as a continuous covariate using restricted cubic splines with 3 df- and we allowed for time-varying effect by including interactions between calendar year and attained age (using splines with 2 df for both).

To obtain estimates of RS for breast cancer and colon cancer patients FPRMs were used, as shown in Eq. (3). The baseline log cumulative excess hazard was modelled smoothly using restricted cubic splines with 5 df. Age at diagnosis was included as a continuous variable using restricted cubic splines with 3 df and we allowed for time-varying effect by including interactions between age and log time (using splines with 2 df and 3 df for age and log time, respectively). The expected mortality rates $h^*(t)$ for each cancer patient at the time of death due to any cause are required as shown in Eq. (4). These expected mortality rates $h^*(t)$ for each age and calendar year were obtained by fitting a Poisson model to the comparators adjusting for attained age and attained year. Predicted mortality rates from this Poisson model were used in the likelihood (4) and assumed to be fixed.

LE_C_, LE_exp_ and LLE by age and year at diagnosis as well as their marginal estimates were obtained with the suggested approach, where the uncertainty in the expected mortality rates was included in the estimation of LE_exp_, LE_C_ and LLE as described above. We refer to it as *modelled w.u*. The estimates obtained with this approach were then compared with the approach, where expected mortality and expected survival are obtained as predictions from the model (8) but the uncertainty from $\widehat{h^*(t)}$ and $\widehat{S^*}(t)$ is not incorporated. This approach is denoted as *modelled w/o u*. For illustrative purposes only, estimates of LE_C_, LE_exp_ and LLE obtained with a conventional approach (*standard*) were also included. In the conventional approach, the life tables of the whole population in Sweden stratified by age, sex and calendar year [22] were used in the estimation of RS and LLE and any uncertainty in the expected measures was ignored.

As a complement to the above-mentioned estimates, conditional and marginal estimates of 15-year restricted mean survival times for both the cancer population and controls and their standard errors were obtained with the same three approaches. 15-year restricted survival time for the cancer population (RMST_C_) quantifies the average life expectancy for cancer patients within the first 15 years since diagnosis.

### Measure of comparison

To compare SEs obtained with the two modelling approaches with and without uncertainty in the expected measures, we estimated the relative % precision (RP):

$$\begin{aligned} RP = 100 \cdot \left( \left( \frac{SE_{\text {modelled w.u.}}}{SE_{\text {modelled w/o u.}}}\right) ^2-1\right) \end{aligned}$$

RP is defined as the percentage disparity in precision when comparing the outcomes of these two modelling approaches. For instance, a RP of 100 % implies that the variance obtained through the modelling approach that incorporates uncertainty is twice as big as the variance obtained through the modelling approach that does not include uncertainty.

All analyses were performed with the publicly available Stata software packages stpm2 and standsurv [9, 19], and all analysis were performed in Stata 17 [23].

## Results

The Point Estimates (PE) of LLE, LE_C_ and LE_exp_ by selected ages at diagnosis (55, 65, 75, 85) and selected years at diagnosis (1992, 1997 and 2002) obtained with the approaches outlined in (“Analysis”) section are presented in Supplementary Table 1 for breast cancer and Supplementary Table 2 for colon cancer. Even though the model for excess mortality does not include year of diagnosis, the LE_exp_, LE_C_ and LLE vary over calendar year since expected mortality differs across calendar year. The Standard Errors (SE) and 95% Confidence Intervals (CIs) for each of the estimates are also shown as well as Relative % Precision (RP), comparing modelling approaches.

**Fig. 1:**
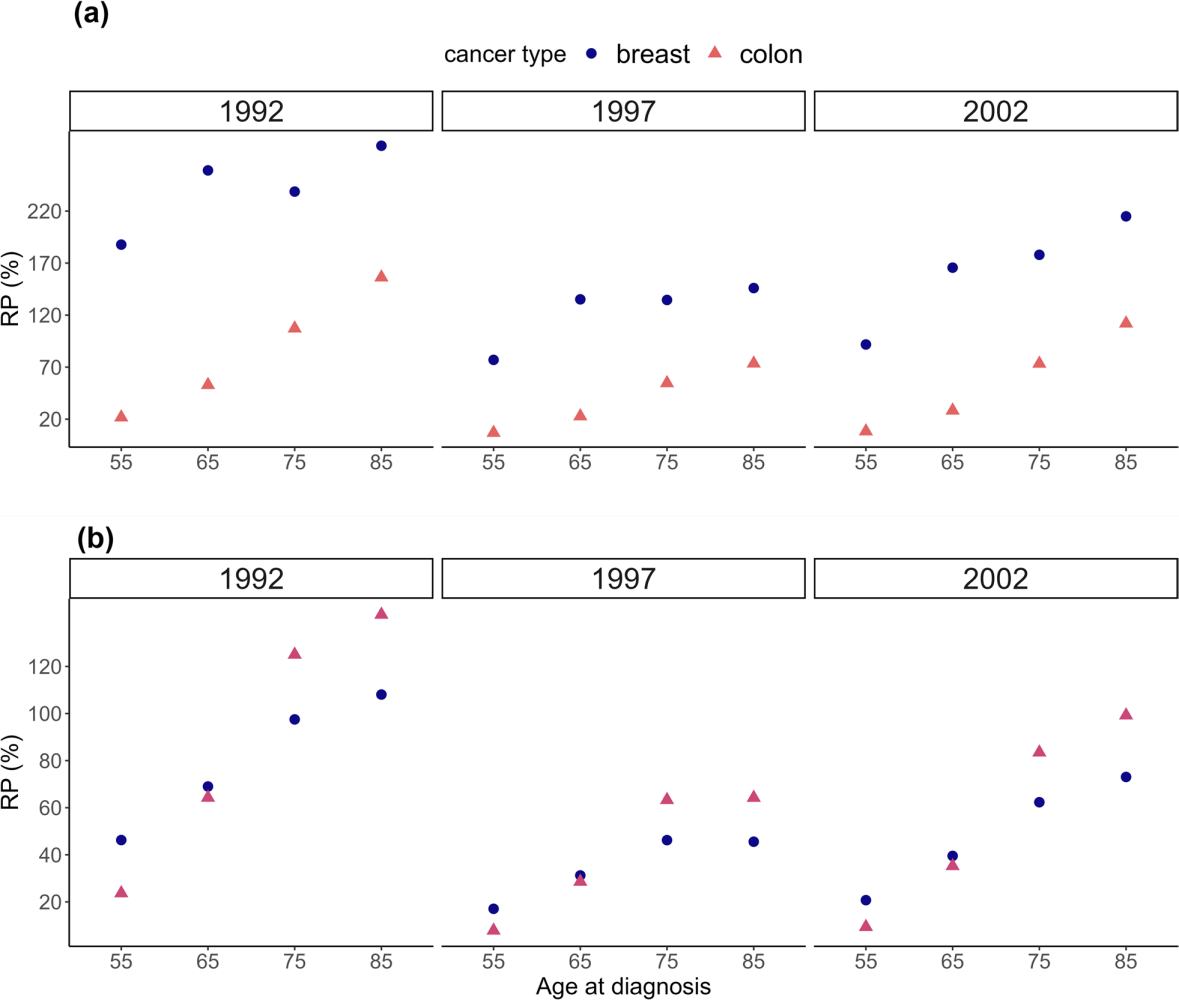
Relative (%) precision (RP) comparing the modelling approaches, where uncertainty in the expected mortality rates used to estimate expected life expectancy, LE_exp_, life expectancy for cancer patients, LE_C_, and loss in life expectancy due to cancer, LLE, is 1) taken into account and 2) ignored. Results are presented for women, aged 55, 65, 75 and 85 years at breast or colon cancer diagnosis in 1992, 1997 and 2002. The above graph (**a**) presents RP of LE_C_ and the below graph (**b**) depicts RP of LLE

Graphical comparisons of the two approaches are presented in Fig. 1. SEs of LLE and LE_C_ obtained with *modelled approach w.u.* were larger than SEs of LLE and LE_C_ obtained with *modelled approach w/o u*. The results were consistent across cancer type, age and year at diagnosis. RP for LE_C_ and LLE generally increased with age. For example, the RP of LLE for females aged 55 years diagnosed with breast cancer in 2002 was approximately 21% while it was approximately 73% for females aged 85 years diagnosed in the same year. For females diagnosed with colon cancer, RPs of LE_C_ were approximately 8% and 112% for patients aged 55 and 85 years, respectively, diagnosed in 2002.

It is noticeable that RP for LE_C_ was higher for breast cancer patients than for colon cancer patients. For instance, RP of LE_C_ for a 65-year-old female diagnosed in 2002 was approximately 166% and 28% for breast and colon cancer, respectively. RPs of LLE were similar for younger patients diagnosed with breast or colon cancer. However, for elderly women diagnosed with breast cancer, RP was smaller than for elderly women diagnosed with colon cancer. For instance, the RP of LLE of a 75-year-old woman diagnosed with breast cancer in 1997 was approximately 46% and for a 75-year-old woman diagnosed with colon cancer in 1997, the RP of LLE was 63%.

Values of RP of LE_C_ were much higher than values of RP for LLE for women diagnosed with breast cancer across all ages and calendar years. For example, the RP of LE_C_ and LLE for females aged 55 years diagnosed with breast cancer in 2002 was approximately 92% and 21%, respectively. For women diagnosed with colon cancer, RP of LLE and RP of LE_C_ were very similar. In particular, the RP of LE_C_ and LLE for females aged 55 years diagnosed with colon cancer in 2002 was approximately 8% and 9%, respectively.

**Fig. 2:**
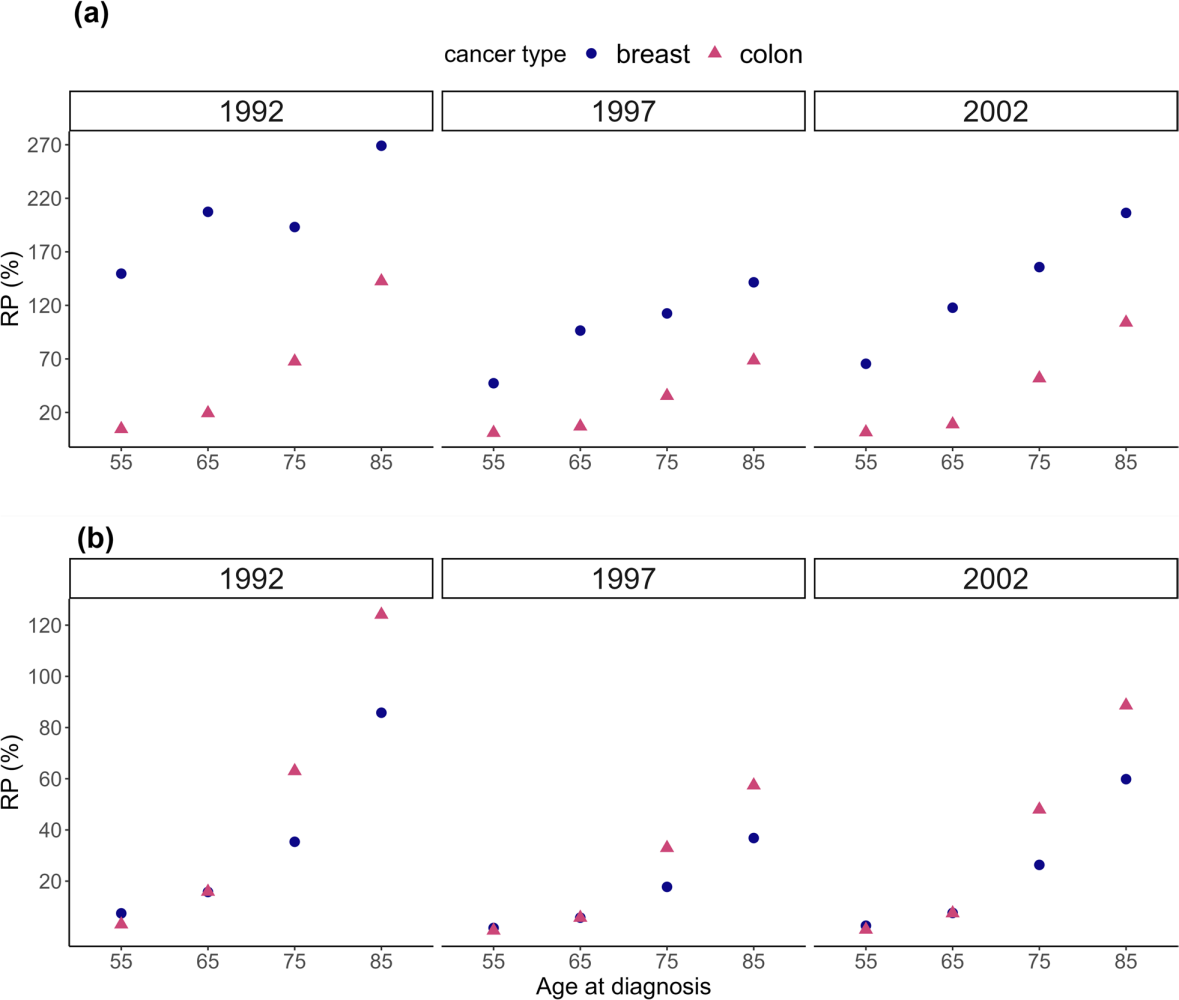
Relative (%) precision (RP) comparing the modelling approaches, where uncertainty in the expected mortality rates used to estimate expected 15-year restricted mean survival time, RMST_exp_, 15-year restricted mean survival time for cancer patients, RMST_C_, and 15-year loss in restricted mean survival time, LRMST, is 1) taken into account and 2) ignored. Results are presented for women, aged 55, 65, 75 and 85 years at breast or colon cancer diagnosis in 1992, 1997 and 2002. The above graph (**a**) presents RP of 15-year RMST_C_ and the below graph (**b**) depicts RP of 15-year LRMST

Similar patterns to the estimates of LE_exp_, LE_C_ and LLE presented in Supplementary Tables 1, 2 and Fig. 1 could be seen for estimates of 15-year RMST in Supplementary Table 3 for breast cancer, Supplementary Table 4 for colon cancer and Fig. 2. The increase in RP of 15-year loss in RMST and 15-year RMST_C_ was seen across all ages. Also, RP of 15-year RMST_C_ was higher for breast cancer patients than for colon cancer patients.

**Fig. 3:**
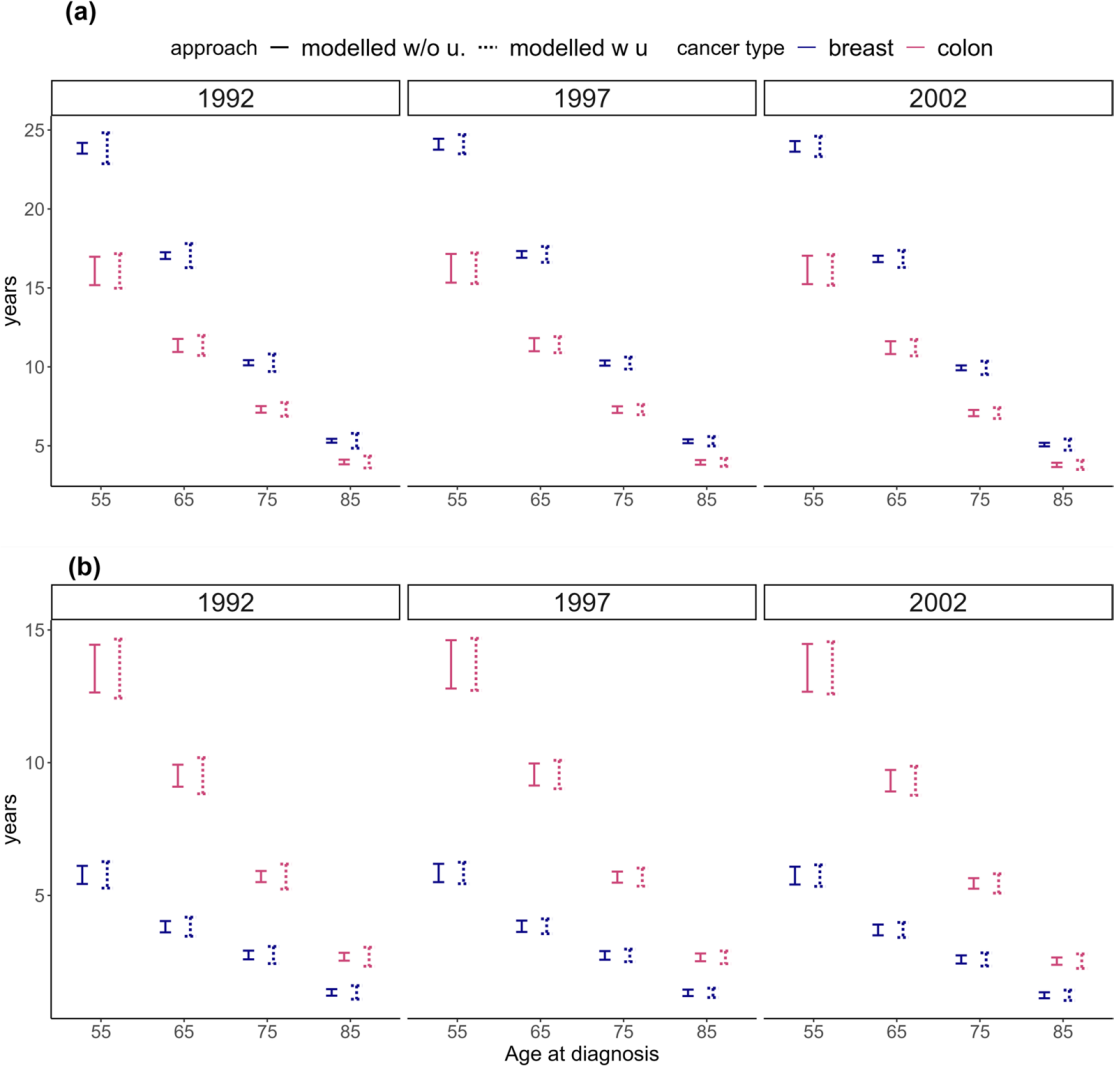
95% confidence intervals of life expectancy LE_C_ (graph (**a**)) and loss in life expectancy LLE (graph (**b**)) for cancer patients from two modelling approaches: 1) when including uncertainty in the expected mortality rates in the estimation (modelled w.u.), and 2) when ignoring uncertainty in the expected mortality rates in the estimation (modelled w/o u.). Results are presented for women, aged 55, 65, 75 and 85 years at breast cancer or colon cancer diagnosis in 1992, 1997 and 2002

**Fig. 4:**
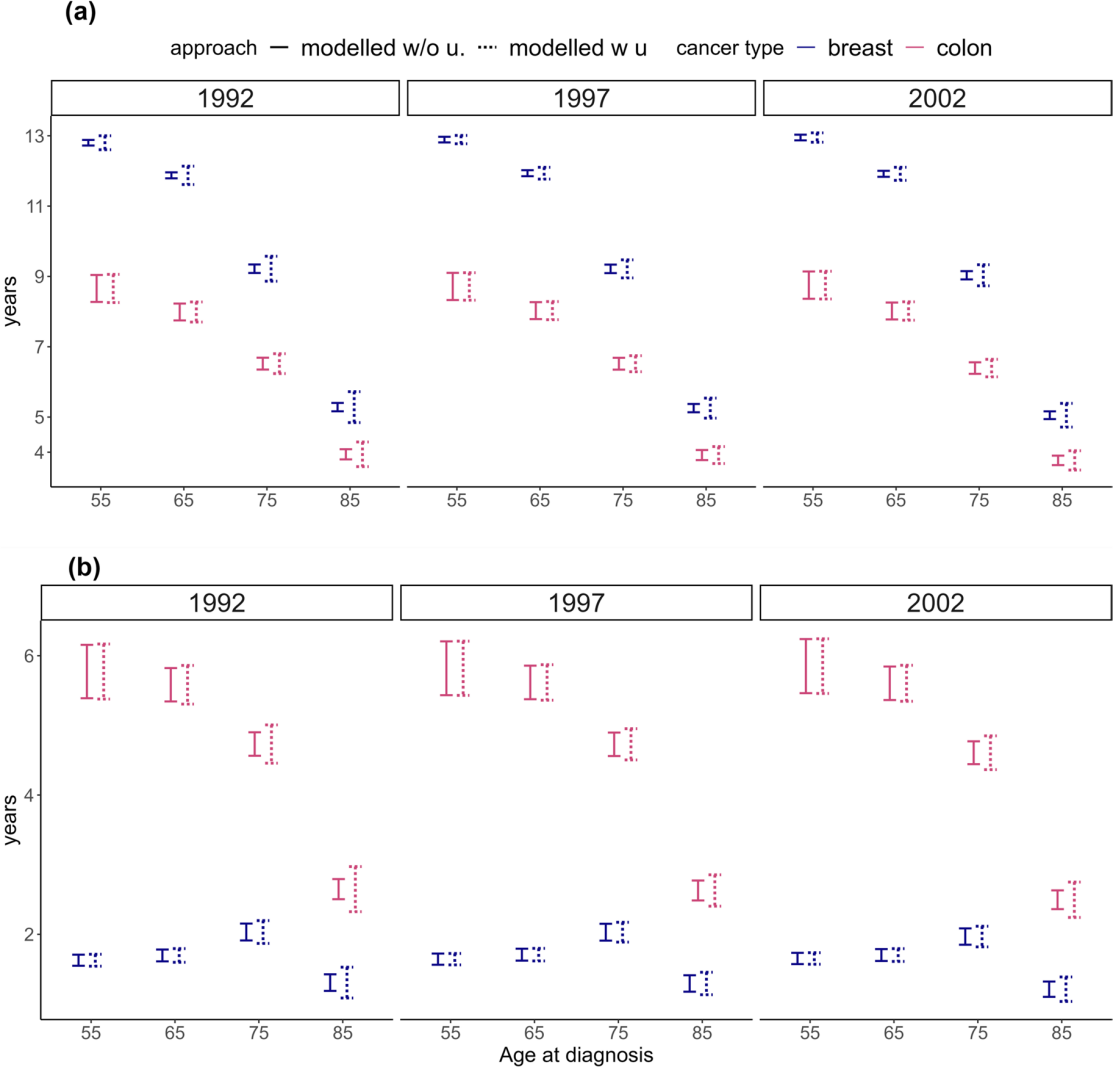
95% confidence intervals of observed 15-year restricted mean survival time, 15-year RMST_C_ (graph (**a**)) and loss in 15-year restricted mean survival time, 15-year LRMST (graph (**b**)) from two modelling approaches: 1) when including uncertainty in the expected mortality rates in the estimation (modelled w.u.), and 2) when ignoring uncertainty in the expected mortality rates in the estimation (modelled w/o u.). Results are presented for women, aged 55, 65, 75 and 85 years at breast cancer or colon cancer diagnosis in 1992, 1997 and 2002

Figures 3 and 4 present 95% CIs of LLE and LE_C_ for modelling approaches with and without including uncertainty in the expected mortality for breast and colon cancer by selected ages at diagnosis (55, 65, 75 and 85 years) and selected years at diagnosis (1992, 1997 and 2002).

Point estimates, standard errors, 95% confidence intervals and relative % precision for marginal LLE, LE_C_ and loss in 15-year RMST for breast and colon cancers obtained with the modelling approaches are illustrated in Table 1. An increase in SE of all estimates obtained with *modelled w.u.* compared to SE obtained with *modelled w/o u.* was seen. For colon cancer, the RP of LE_C_ was almost the same as the RP of LLE, around 50%. In contrast, for breast cancer, the RP for LE_C_ (179%) was almost 5 times bigger than the RP of LLE (34%).

**Table 1:** Point estimates (PE) of marginal loss in life expectancy (LLE), life expectancy (LE_C_) and expected life expectancy (LE_exp_) for cancer patients together with PE of marginal loss in 15-year restricted mean survival times (LRMST), observed 15-year restricted mean survival time (RMST_C_) and expected 15-year restricted mean survival time (RMST_exp_) for cancer patients, with standard errors (SE), relative % precision (RP), lower (LCI) and upper (UCI) 95% confidence intervals. RP illustrates the comparison of the modelling approaches. All the estimates are measured in years

**Approach**	PE	SE	RP(%)	LCI	UCI	PE	SE	RP(%)	PE	SE
	Loss in Life Expectancy (LLE)					Life Expectancy for cancer patients (LE_C_)			Expected Life Expectancy (LE_exp_)	
	**breast cancer**									
modelled w/o u	3.94	0.082		3.78	4.10	16.22	0.082		20.16	
modelled w.u.	3.94	0.095	34.14	3.75	4.13	16.22	0.137	178.83	20.16	0.157
	**colon cancer**									
modelled w/o u	6.36	0.091		6.18	6.54	8.02	0.091		14.38	
modelled w.u.	6.36	0.114	57.88	6.14	6.58	8.02	0.111	50.47	14.38	0.134
	Loss in 15-year restricted mean survival time (LRMST)					Observed 15-year RMST_C_			Expected 15-year RMST_exp_	
	**breast cancer**									
modelled w/o u	1.70	0.028		1.64	1.75	10.67	0.028		12.36	
modelled w.u.	1.70	0.030	11.18	1.64	1.75	10.67	0.043	135.75	12.36	0.042
	**colon cancer**									
modelled w/o u	4.36	0.058		4.24	4.47	6.23	0.058		10.59	
modelled w.u.	4.36	0.065	25.85	4.23	4.48	6.23	0.066	29.96	10.59	0.061

## Discussion

The main purpose of this paper is to propose an approach for including the uncertainty of the expected mortality rates in the estimation of life years remaining since diagnosis (LE_C_) and loss in life expectancy (LLE) for cancer patients in a situation when life tables based on the entire general population are unavailable, and instead, a sample from the general population is utilised.

Aiming to validate the necessity of the suggested approach, we illustrated that standard errors (SE) of LE_C_ and LLE obtained with the suggested approach were larger than SE of LE_C_ and LLE, obtained with the assumption of known (fixed) mortality rates from the general population.

For younger patients diagnosed with cancer, cancer-specific mortality usually prevails over other-cause mortality; thus, the variance in the expected mortality rates might become negligible. However, cancer patients tend to be old, and there will be competing causes of death other than cancer. In such a case, ignoring the population component can lead to a substantial underestimation of the variances of LE_C_ and LLE, and thus, much narrower confidence intervals. In this study, the variance of LLE (LE_C_), for instance, for females diagnosed with breast cancer at 65 and 85 years old in 2002 obtained with the suggested approach were 40% (166%) and 73% (215%), respectively, larger than variance of LLE (LE_C_) obtained with the approach without including uncertainty in the expected measures.

For different cancer types, other-cause mortality can prevail over cancer-specific mortality at different times since diagnosis. In this study, we have presented estimates of the variance of LE_C_ and LLE for colon and breast cancer. Colon cancer is characterised by higher excess mortality than breast cancer, where longer survival is more common. The variance for LE_C_, for example, for females diagnosed at 55 years old in 2002 with breast and colon cancer were 92% and 8%, respectively, larger compared to the variance obtained with the approach when variation in the expected measures was ignored .

The estimation of LLE includes uncertainty in the expected mortality rates in the estimation of both LE_exp_ and LE_C_. This will influence the extent of the underestimation of the variance of LLE. We can expect more severe underestimation for LE_C_ than LLE. The marginal estimates of the variances of LLE (LE_C_) showed larger differences. Variances of marginal LLE (LE_C_) for females diagnosed with breast and colon cancer obtained with the modelling approach including uncertainty in the expected measures were 34% (179%) and 58% (50%) larger, respectively, than variances of marginal LLE (LE_C_) obtained with the modelling approach ignoring uncertainty in the expected measures.

We have provided an approach to include uncertainty of the expected mortality rates in the estimation of LE_exp_, LE_C_ and LLE. The question of the variance of non-parametric LLE has been discussed in a previous paper [24]. However, a bootstrap approach was suggested for estimating the variance, which can be a time-consuming with big data sets and especially for marginal measures. In this paper, we used flexible parametric relative survival models to obtain the variances of the expected life expectancy for cancer patients if they did not have cancer, LE_exp_, life expectancy for cancer patients, LE_C_, and the loss in life expectancy, LLE, for cancer patients in comparison with the general population. Another advantage of the suggested approach is the usage of existing Stata software and an example of Stata code is included. This makes it easy to implement the approach in various research projects. However, it is essential to acknowledge that the suggested approach has a limitation as it does not consider the variation of expected mortality rates when estimating relative survival. This can be a possible extension of the suggested approach. This study exclusively focused on conducting an empirical assessment of the suggested approach. However, a comprehensive simulation study could offer additional insights into the new approach’s performance across various scenarios.

There are also other issues in our work which were not explored here, but which could be of possible interest. In this study we utilised comparators for cancer patients to estimate expected values for cancer patients if they did not have cancer. In cases where a comparable sample is unavailable, the modelling process can be used by directly adjusting the available background population, as discussed in previous research [25–27]. Nevertheless, although those proposed models are valuable in addressing non-comparability bias, they do not account for potential variability in expected mortality rates. For example, Touraine et al. [26] proposed a model, which becomes unstable by allowing the background mortality to change, even though, it was observed that model estimates’ variability increased with the inclusion of corrective parameters. Additionally, the model featuring a random effect, as proposed by Rubio et al. [25], is not recommended for data sets with fewer than 5,000 observations or with a censoring rate exceeding 50%. To address these limitations, further research is necessary to incorporate a possible uncertainty in the estimates of the expected measures in these models.

In conclusion, by accounting for the uncertainty of expected mortality rates, the proposed method ensures accurate estimates of the variance of LE_C_ and LLE for cancer patients when a sample from the general population is used. This is particularly valuable for older patients and cancer types with longer survival time, where underestimation of the variance can be substantial when the entire general population data are not accessible.

### Supplementary information

**Additional file 1.**

**Additional file 2.**

**Additional file 3.**

**Additional file 4.**

**Additional file 5.**

**Additional file 6.**

**Additional file 7.**

## Data Availability

The data used for this study may not, according to the ethical permission granted for its use, be shared by the authors to a third party. It is accessible by application to the Swedish authorities (The Swedish Cancer Registry).
