# Supplementary material for: Including uncertainty of the expected mortality rates in the prediction of loss in life expectancy
Source: BMC Med Res Methodol. 2023 Dec 12;23:291. doi: 10.1186/s12874-023-02118-w (PMC10714581; doi:10.1186/s12874-023-02118-w)
Supplement: Supplementary file 2 — Additional file 2. [file 12874_2023_2118_MOESM2_ESM.pdf]

Table S2: Point estimates (PE) with lower (LCI) and upper (UCI) 95% confidence intervals, standard errors (SE) and relative % precision (RP) of Loss in Life Expectancy (LLE), Life Expectancy for cancer patients (LE_C) and their Life Expectancy if they did not have cancer (LE_{exp}), obtained with different approaches. Results are presented for women, aged 55, 65, 75 and 85 years at colon cancer diagnosis in 1992, 1997 and 2002 years. All PEs are measured in years. RP illustrates the comparison of modelling approaches with and without uncertainty in the expected measures.

Approach	Age at diagnosis	**Expected Life Expectancy (LE_{exp})**		**Life Expectancy for cancer patients (LE_C)**			**Loss in Life expectancy (LLE)**				
		PE	SE	PE	SE	RP (%)	PE	SE	RP (%)	LCI	UCI
				**diagnosed in 1992**							
modelled w.u.	55	29.62	0.65	16.08	0.56	21.73	13.54	0.57	23.69	12.43	14.66
modelled w/o u.	55	29.62		16.08	0.46		13.54	0.46		12.64	14.44
standard	55	29.99		16.59	0.48		13.40	0.48		12.47	14.33
modelled w.u.	65	20.87	0.52	11.36	0.32	52.91	9.51	0.35	64.28	8.83	10.19
modelled w/o u.	65	20.87		11.36	0.21		9.51	0.21		9.10	9.92
standard	65	20.60		11.38	0.21		9.22	0.21		8.80	9.64
modelled w.u.	75	13.02	0.41	7.31	0.22	107.25	5.71	0.24	125.03	5.24	6.18
modelled w/o u.	75	13.02		7.31	0.11		5.71	0.11		5.50	5.92
standard	75	12.09		7.04	0.10		5.05	0.10		4.85	5.24
modelled w.u.	85	6.67	0.34	3.98	0.19	156.28	2.69	0.18	142.01	2.33	3.05
modelled w/o u.	85	6.67		3.98	0.08		2.69	0.08		2.54	2.84
standard	85	6.04		3.75	0.07		2.29	0.07		2.16	2.43
				**diagnosed in 1997**							
modelled w.u.	55	29.95	0.36	16.24	0.50	6.92	13.70	0.50	7.83	12.72	14.68
modelled w/o u.	55	29.95		16.24	0.46		13.70	0.46		12.79	14.61
standard	55	30.41		16.80	0.48		13.61	0.48		12.66	14.56
modelled w.u.	65	20.96	0.32	11.41	0.26	22.80	9.56	0.27	28.59	9.02	10.09
modelled w/o u.	65	20.96		11.41	0.21		9.56	0.21		9.14	9.97
standard	65	21.01		11.58	0.22		9.43	0.22		9.00	9.86
modelled w.u.	75	12.98	0.26	7.29	0.16	54.64	5.69	0.17	63.30	5.35	6.03
modelled w/o u.	75	12.98		7.29	0.11		5.69	0.11		5.48	5.90
standard	75	12.53		7.27	0.11		5.26	0.11		5.06	5.47
modelled w.u.	85	6.62	0.20	3.95	0.13	73.54	2.67	0.12	64.24	2.43	2.91
modelled w/o u.	85	6.62		3.95	0.08		2.67	0.08		2.52	2.81
standard	85	6.18		3.83	0.07		2.35	0.07		2.21	2.49
				**diagnosed in 2002**							
modelled w.u.	55	29.71	0.40	16.14	0.50	8.33	13.57	0.50	9.38	12.59	14.56
modelled w/o u.	55	29.71		16.14	0.46		13.57	0.46		12.67	14.47
standard	55	30.64		16.92	0.49		13.72	0.49		12.77	14.68
modelled w.u.	65	20.54	0.35	11.22	0.27	28.34	9.32	0.28	35.23	8.77	9.87
modelled w/o u.	65	20.54		11.22	0.21		9.32	0.21		8.92	9.73
standard	65	21.47		11.81	0.23		9.66	0.23		9.22	10.11
modelled w.u.	75	12.53	0.30	7.07	0.18	73.39	5.45	0.19	83.49	5.09	5.82
modelled w/o u.	75	12.53		7.07	0.10		5.45	0.10		5.25	5.65
standard	75	12.95		7.48	0.11		5.47	0.11		5.25	5.68
modelled w.u.	85	6.32	0.25	3.79	0.15	112.02	2.53	0.14	99.23	2.25	2.80
modelled w/o u.	85	6.32		3.79	0.07		2.53	0.07		2.39	2.66
standard	85	6.35		3.93	0.07		2.43	0.07		2.28	2.57
