# Supplementary material for: Including uncertainty of the expected mortality rates in the prediction of loss in life expectancy
Source: BMC Med Res Methodol. 2023 Dec 12;23:291. doi: 10.1186/s12874-023-02118-w (PMC10714581; doi:10.1186/s12874-023-02118-w)
Supplement: Supplementary file 4 — Additional file 4. [file 12874_2023_2118_MOESM4_ESM.pdf]

Table S4: Point estimates (PE) with lower (LCI) and upper (UCI) 95% confidence intervals, standard errors (SE) and relative % precision (RP) of an expected 15-year restricted mean survival time (15-year $RMST_{exp}$), observed 15-year restricted mean survival time (15-year $RMST_C$) and loss in 15-year restricted mean survival time (15-year LRMST), obtained with different approaches. Results are presented for women, aged 55, 65, 75 and 85 years at diagnosis being diagnosed with colon cancer in 1992, 1997 and 2002 years. All PEs are measured in years. RP illustrates the comparison of modelling approaches with and without uncertainty in the expected measures.

Approach	Age at diagnosis	**Expected 15-year $RMST_{exp}$**		**Observed 15-year $RMST_C$**			**Loss in 15-year restricted mean survival time (LRMST)**				
		PE	SE	PE	SE	RP (%)	PE	SE	RP (%)	LCI	UCI
				**diagnosed in 1992**							
modelled w.u.	55	14.43	0.11	8.66	0.21	4.54	5.77	0.20	3.12	5.38	6.17
modelled w/o u.	55	14.43		8.66	0.20		5.77	0.20		5.39	6.16
standard	55	14.37		8.70	0.20		5.68	0.20		5.29	6.06
modelled w.u.	65	13.57	0.15	7.99	0.15	19.39	5.58	0.14	15.88	5.31	5.86
modelled w/o u.	65	13.57		7.99	0.12		5.58	0.12		5.34	5.82
standard	65	13.41		7.94	0.12		5.47	0.12		5.23	5.71
modelled w.u.	75	11.25	0.23	6.52	0.14	67.66	4.73	0.14	63.01	4.46	5.01
modelled w/o u.	75	11.25		6.52	0.09		4.73	0.09		4.56	4.90
standard	75	10.62		6.32	0.08		4.30	0.08		4.14	4.46
modelled w.u.	85	6.59	0.31	3.94	0.18	142.58	2.65	0.17	124.12	2.32	2.97
modelled w/o u.	85	6.59		3.94	0.07		2.65	0.07		2.50	2.79
standard	85	5.96		3.71	0.07		2.26	0.07		2.13	2.39
modelled w.u.	55	14.53	0.05	8.71	0.20	1.04	5.82	0.20	0.71	5.43	6.21
				**diagnosed in 1997**							
modelled w/o u.	55	14.53		8.71	0.20		5.82	0.20		5.43	6.21
standard	55	14.40		8.71	0.20		5.69	0.20		5.30	6.07
modelled w.u.	65	13.64	0.09	8.03	0.13	6.95	5.62	0.13	5.72	5.36	5.87
modelled w/o u.	65	13.64		8.03	0.12		5.62	0.12		5.38	5.86
standard	65	13.52		8.00	0.12		5.52	0.12		5.28	5.76
modelled w.u.	75	11.25	0.15	6.52	0.12	35.52	4.73	0.11	32.98	4.50	4.95
modelled w/o u.	75	11.25		6.52	0.09		4.73	0.09		4.56	4.90
standard	75	10.89		6.47	0.09		4.43	0.09		4.26	4.59
modelled w.u.	85	6.55	0.19	3.92	0.12	68.66	2.63	0.12	57.43	2.40	2.85
modelled w/o u.	85	6.55		3.92	0.07		2.63	0.07		2.49	2.77
standard	85	6.10		3.78	0.07		2.32	0.07		2.18	2.45
				**diagnosed in 2002**							
modelled w.u.	55	14.60	0.06	8.75	0.20	1.53	5.85	0.20	1.06	5.46	6.24
modelled w/o u.	55	14.60		8.75	0.20		5.85	0.20		5.46	6.24
standard	55	14.45		8.74	0.20		5.71	0.20		5.32	6.10
modelled w.u.	65	13.62	0.10	8.02	0.13	8.98	5.60	0.13	7.46	5.35	5.86
modelled w/o u.	65	13.62		8.02	0.12		5.60	0.12		5.36	5.84
standard	65	13.64		8.06	0.13		5.58	0.13		5.34	5.82
modelled w.u.	75	11.00	0.19	6.39	0.13	51.91	4.61	0.12	47.99	4.36	4.85
modelled w/o u.	75	11.00		6.39	0.08		4.61	0.08		4.44	4.77
standard	75	11.13		6.59	0.09		4.54	0.09		4.37	4.71
modelled w.u.	85	6.26	0.23	3.77	0.14	103.95	2.50	0.13	88.66	2.24	2.75
modelled w/o u.	85	6.26		3.77	0.07		2.50	0.07		2.36	2.63
standard	85	6.26		3.87	0.07		2.38	0.07		2.24	2.52
