# Supplementary material for: Including uncertainty of the expected mortality rates in the prediction of loss in life expectancy
Source: BMC Med Res Methodol. 2023 Dec 12;23:291. doi: 10.1186/s12874-023-02118-w (PMC10714581; doi:10.1186/s12874-023-02118-w)
Supplement: Supplementary file 7 — Additional file 7. [file 12874_2023_2118_MOESM7_ESM.pdf]

Estimation of variance of LLE

$\mathrm{LE_{exp}}$ and $\mathrm{LE_C}$ are asymptotically normal random variables, so their difference LLE is also random variable with $N(\mu;\Sigma)$, where the expectation $\mu = E(LLE) = E(LE_{\mathrm{exp}}) - E(LE_C)$ and variance-covariance matrix Σ can be found by matrix multiplication:

$$\Sigma = G^T \cdot V \cdot G, \tag{1}$$

where

$$G = \left(\frac{\partial(LE_{\mathrm{exp}} - LE_C)}{\partial\widehat{\beta}}\right) = \begin{pmatrix} \frac{\partial(LE_{\mathrm{exp}} - LE_C)}{\partial\widehat{\beta_{21}}} \\ \frac{\partial(LE_{\mathrm{exp}} - LE_C)}{\partial\widehat{\beta_{22}}} \\ \dots \\ \frac{\partial(LE_{\mathrm{exp}} - LE_C)}{\partial\widehat{\beta_{2p_2}}} \\ -\frac{\partial LE_C}{\partial\widehat{\beta_{11}}} \\ \dots \\ -\frac{\partial LE_C}{\partial\widehat{\beta_{1p_1}}} \end{pmatrix}, \tag{2}$$

where p_1 is the number of parameters in vector $\boldsymbol{\beta_1}$, p_2 is the number of parameters in vector $\boldsymbol{\beta_2}$ and $\boldsymbol{\beta}$ is a vector of $p = p_1 + p_2$ parameters:

$$\widehat{\beta} = \left(\widehat{\beta_2}\ \widehat{\beta_1}\right)^T = \left(\widehat{\beta_{21}}\ \widehat{\beta_{22}}\ \dots\ \widehat{\beta_{2p_2}}\ \widehat{\beta_{11}}\ \widehat{\beta_{12}}\ \dots\ \widehat{\beta_{1p_1}}\right)^T$$

$$\frac{\partial\widehat{LE_C}}{\partial\widehat{\beta}} = -\frac{\partial\int_0^{t^*}\widehat{Cr_{cancer}(u)du}}{\partial\widehat{\beta}} - \frac{\partial\int_0^{t^*}\widehat{Cr_{other}(u)du}}{\partial\widehat{\beta}}$$
$$= -\frac{\partial\int_0^{t^*}\widehat{R(u)}\cdot\widehat{S^*(a_0+u)}\cdot\widehat{h^*(a_0+u)}du}{\partial\widehat{\beta}} - \frac{\partial\int_0^{t^*}\widehat{R(u)}\cdot\widehat{S^*(a_0+u)}\cdot\widehat{\lambda(u)}du}{\partial\widehat{\beta}}$$

and

$$\frac{\partial\widehat{LE_{\mathrm{exp}}}}{\partial\widehat{\beta}} = \frac{\partial\int_0^{t^*}\widehat{S^*(a_0+u)}du}{\partial\widehat{\beta}} = \left(\frac{\partial\int_0^{t^*}\widehat{S^*(a_0+u)}}{\partial\widehat{\beta_{21}}}\ \frac{\partial\int_0^{t^*}\widehat{S^*(a_0+u)}}{\partial\widehat{\beta_{22}}}\ \dots\ \frac{\partial\int_0^{t^*}\widehat{S^*(a_0+u)}}{\partial\widehat{\beta_{2p_2}}}\ 0_1\ \dots\ 0_{p_1}\right)$$

Variance-covariance $p \times p$ matrix V can be presented as:

$$V = \begin{pmatrix} \sigma^2_{\beta_{21}} & \sigma_{\beta_{21},\beta_{22}} & ... & \sigma_{\beta_{21},\beta_{2p_2}} & 0 & 0 & ... & 0 \\ \sigma_{\beta_{22},\beta_{21}} & \sigma^2_{\beta_{22}} & ... & \sigma_{\beta_{22},\beta_{2p_2}} & 0 & 0 & ... & 0 \\ ... & ... & ... & ... & ... & ... & ... & \\ \sigma_{\beta_{2p_2},\beta_{21}} & \sigma_{\beta_{2p_2},\beta_{22}} & ... & \sigma^2_{\beta_{2p_2}} & 0 & 0 & ... & 0 \\ 0 & 0 & ... & 0 & \sigma^2_{\beta_{11}} & \sigma_{\beta_{11},\beta_{12}} & ... & \sigma_{\beta_{11},\beta_{1p_1}} \\ 0 & 0 & ... & 0 & \sigma_{\beta_{11},\beta_{12}} & \sigma^2_{\beta_{12}} & ... & \sigma_{\beta_{12},\beta_{1p_1}} \\ ... & ... & ... & ... & ... & ... & ... & ... \\ 0 & 0 & ... & 0 & \sigma_{\beta_{1p_1},\beta_{11}} & \sigma_{\beta_{1p_1},\beta_{12}} & ... & \sigma^2_{\beta_{1p_1}} \end{pmatrix}, \quad (3)$$

where $V_2(\hat{\beta_2})$ is the variance-covariance matrix from model for expected mortality:

$$V_2(\hat{\beta_2}) = \begin{pmatrix} \sigma^2_{\beta_{21}} & \sigma_{\beta_{21},\beta_{22}} & ... & \sigma_{\beta_{21},\beta_{2p_2}} \\ \sigma_{\beta_{22},\beta_{21}} & \sigma^2_{\beta_{22}} & ... & \sigma_{\beta_{22},\beta_{2p_2}} \\ ... & & & \\ \sigma_{\beta_{2p_2},\beta_{21}} & \sigma_{\beta_{2p_2},\beta_{22}} & ... & \sigma^2_{\beta_{2p_2}} \end{pmatrix} \quad (4)$$

Variance-covariance matrix $V_1(\hat{\beta_1})$ from model for excess mortality is then:

$$V_1(\hat{\beta_1}) = \begin{pmatrix} \sigma^2_{\beta_{11}} & \sigma_{\beta_{11},\beta_{12}} & ... & \sigma_{\beta_{11},\beta_{1p_1}} \\ \sigma_{\beta_{11},\beta_{12}} & \sigma^2_{\beta_{12}} & ... & \sigma_{\beta_{12},\beta_{1p_1}} \\ ... & & & \\ \sigma_{\beta_{1p_1},\beta_{11}} & \sigma_{\beta_{1p_1},\beta_{12}} & ... & \sigma^2_{\beta_{1p_1}} \end{pmatrix} \quad (5)$$
